# Diagnosis of 65 cases of ampullary renal pelvis after postnatal follow-up of 1,167 newborn infants with prenatally suspected hydronephrosis

**DOI:** 10.3892/etm.2014.2076

**Published:** 2014-11-18

**Authors:** LEI ZHANG, CHAO LIU, FUJIANG LI, XIANG LI, CHAO SUN, HAO SUN

**Affiliations:** Department of Pediatric Surgery, Qilu Hospital, Shandong University, Jinan, Shandong 250012, P.R. China

**Keywords:** hydronephrosis, ampullary renal pelvis, follow-up studies

## Abstract

The aim of the present study was to assess the morbidity of ampullary renal pelvis (ARP) and document its natural history in post-natal life. A total of 1,167 newborn infants with prenatally suspected hydronephrosis were retrospectively analyzed. Of these, 65 patients were diagnosed with ARP by computed tomography urography (CTU) and/or magnetic resonance urography (MRU). All cases were followed up with ultrasonogrophy at 1, 3, 6 and 12 months after birth, and one case was followed up for 5 years. Changes in the separation of the renal pelvis collection system were recorded. Children with ARP accounted for 5.57% of the total cases (65/1,167) followed-up. No lack of connection between the renal calyces and the renal pelvis was detected. The long-term follow-up revealed that the separation of the renal pelvis collection system did not tend to increase over time. In addition to imaging examinations, long-term follow-up observation is recommended for the accurate diagnosis of pediatric ARP, particularly for differentiation from hydronephrosis.

## Introduction

In recent years, with the improvement of imaging techniques and prenatal care systems, a growing number of cases of pediatric hydronephrosis have been detected in the early prenatal period and followed up (1,2). In addition, an increasing number of cases of pediatric ampullary renal pelvis (ARP), which is challenging to distinguish from hydronephrosis, have been diagnosed in postnatal follow-up observations (3). To improve the diagnostic accuracy of this type of pelvis and to reduce misdiagnosis, the present study reports the findings in the clinical follow-up of 65 cases of ARP from 1,167 pediatric patients who were prenatally suspected to have hydronephrosis.

## Patients and methods

### Patient characteristics

This study retrospectively analyzed 1,167 cases of prenatally suspected hydronephrosis, who were referred to comprehensive pediatric surgery, obstetrics, ultrasound and other related departments in the Qilu Hospital of Shandong University (Jinan, China) between January 2003 and December 2011. For the purpose of this study, different levels of postnatal follow-up program were performed for these cases according to the grade of hydronephrosis. A total of 65 cases were diagnosed with pediatric ARP by complete physical examination by ultrasound, computed tomography urography (CTU) and magnetic resonance imaging of the urinary system (MRU). Of these cases of ARP, 37 were males and 28 were females. The average age of the cases ranged from 2 to 8 years (mean age, 3.9 years). Unilateral and bilateral ARP were diagnosed in 54 (left in 31 and right in 23 cases) and 11 cases, respectively.

### Follow-up surveillance

**Figure 1:**
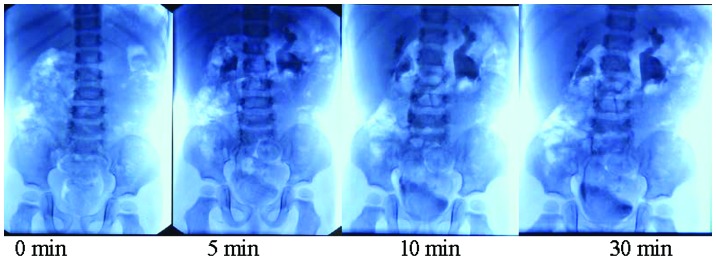
Intravenous pyelography at different time points.

**Figure 2:**
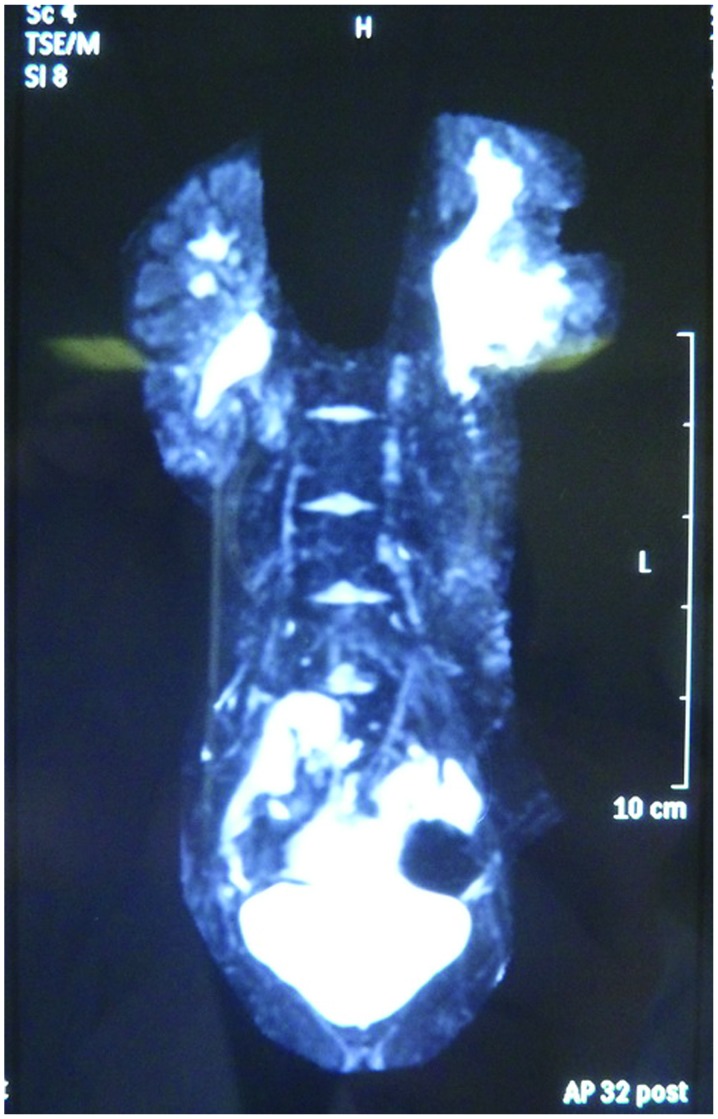
Magnetic resonance imaging.

In the large-scale prenatal screening and follow-up of postnatal hydronephrosis, ARP initially did not show any difference from hydronephrosis by sonographic examination. Ultrasonic examination reported a volatile separation of the renal pelvis collection system by 2–3 cm, with or without mild renal calyx dilatation. Such cases were closely followed up according to the grade of hydronephrosis and ultrasound tests were performed once every one to three months. Compared with cases of ordinary uteropelvic junction (UPJ) obstruction hydronephrosis and pyelonephritis, the separation of the collection system in such cases was always volatile (sometimes large and sometimes small) as the number of follow-up observations increased. Moreover, the calyx in these cases did not show clear dilatation or exhibited no dilatation, and did not tend to increase in the follow-up observation. As the follow-up observations continued, and the in-depth understanding of the disease increased, the cases were identified and summarized. Further imaging studies, such as CTU and MRU, documented the morphology of the renal pelvis in these cases, which were grouped separately from other hydronephrosis cases (Figs. 1 and 2). This group of cases continued to be followed up by ultrasound test every three months and the separation of the renal pelvis collection system was recorded. All these experiments were conducted after informed consent was given by the parents and under the approval by the ethics committee of the Qilu Hospital of Shandong University (Shandong, China).

## Results

The diagnosis of pediatric ARP was made on the basis of imaging examination in the postnatal follow-up observation. Ultrasonic diagnosis showed that the separation of the collection system was mostly volatile by 2–3 cm with only small changes in the next two or three follow-up observations. Even though the hydronephrosis in these cases was mainly Society for Fetal Urology (SFU) classification 2 or 3 (4), CTU or MRU examination showed dilatation of the renal pelvis collection system, but mild renal caliectasis and complete cup-shaped calyx morphology. In total, 37 cases were followed up and diagnosed with ARP by ultrasonic examination (Table I).

## Discussion

In the past, pediatric hydronephrosis was identified when patients received abdominal ultrasound screening due to abdominal mass and abdominal pain. Now, pediatric hydronephrosis can be diagnosed prenatally (5) and closely followed up in postnatal examination (1,6,7). Follow-up program development is based on the hydronephrosis classification (8). With an increase in the follow-up observation and in-depth understanding of pediatric hydronephrosis, pediatric ARP may be detected and verified.

The morphology of the renal pelvis does not have uniform standards in anatomy and imaging. Anatomically, the renal pelvis is situated in the renal sinus and comprises two or three major renal calyces formed from two or three renal calyces. Renal angiography intuitively displays the morphology and type of renal pelvis. The renal pelvis can be divided into intrarenal and renal pelvis according to the location of the pelvis, while the shape of the renal pelvis can be divided into horn, branching, ampullary and transitional types. X-ray angiographic observation demonstrates that ARP is large and round and may be connected with renal calyces instead of major renal calyces.

To the best of our knowledge, this is the first detailed report on the diagnosis of pediatric ARP. With improvements of imaging techniques and prenatal care systems, pediatric UPJ hydronephrosis can be diagnosed and closely followed up in the early fetal period. Therefore, pediatric ARP can be observed in the early postnatal follow-up examination and diagnosed in the continuous follow-up observations. Pediatric hydronephrosis is divided into physiological and pathological pediatric hydronephrosis, which can be diagnosed during postnatal follow-ups. Pediatric ARP can be distinguished from hydronephrosis since in ARP the separation of the renal pelvis collection system does not tend to increase during the postnatal follow-up observation. Wang *et al* reported that by ultrasonic examination, the morphology of the pediatric ampullary pelvis exhibits four shapes, specifically obtuse triangle, shuttle type, oval and dendritic (9). Ultra-sonography reveals that the obtuse triangle and oval ampullary types are mainly characterized by a mild separation of the pelvis collection system with <2 cm width, in the shape of an obtuse triangle or oval. Moreover, the calyx echo is indistinguishable and without a sense of tension; also, no clear changes in the non-echo range are detected in several reviews following urination. The morphology of the shuttle-type pediatric ampullary pelvis is very similar to the physiological separation of the renal sinus, whereas the dendritic ampullary pelvis has a branch-like shape.

Although the ampullary pelvis has previously been described, the clinical manifestations and diagnosis of pediatric ampullary pelvis have not been reported. With the large-scale screening of cases of fetal hydronephrosis and follow-up observation, cases of pediatric ampullary pelvis may be distinguished from those of fetal hydronephrosis. The diagnosis of pediatric ampullary pelvis not only relies on comprehensive analysis of ultrasonography, CTU, MRU and other imaging examinations, but also on long-term dynamic follow-up observation. A full understanding of the pediatric ampullary pelvis is significant to the differential diagnosis of this normal physiological pelvis morphology from pediatric hydronephrosis. To reduce misdiagnosis, it is recommended that renal ultrasound tests in each postnatal follow-up observation are combined with history and previous follow-up test results. It is also recommended that clinicians highly regard complete follow-up observation data to distinguish pediatric ampullary pelvis from pediatric hydronephrosis and make a correct diagnosis and use the correct treatment protocols.

In conclusion, an increasing number of cases of hydronephrosis may be detected ante partum, and patients should be closely followed up until the infant and juvenile periods. The cases may be distinguished by ultrasonography. Thus, the diagnosis methods and timing of the surgical treatment that should be adopted differ from those previously. In addition, it is necessary to distinguish between physiological and pathological hydronephrosis. Hydronephrosis requires close follow-up and appropriate treatment. Finally, selecting an appropriate opportunity for surgical treatment is dependent on the grade of severity, results of follow-up and renal function.

## Figures and Tables

**Table I:** Separation of the renal pelvis and renal cortical thickness as evaluated by ultrasound (mm, mean ± standard deviation).

	Age				
	
Variable	6 days	42 days	3 months	6 months	1 year
Pelvis collection system	22.3±5.60	24.4±6.03	25.5±6.22	23.2±6.3	24.4±5.23
Renal cortical thickness	0.76±0.13	0.69±0.16	0.75±0.17	0.85±0.18	0.75±0.16
